# Degradation of Intrinsically Disordered Proteins by the NADH 26S Proteasome

**DOI:** 10.3390/biom10121642

**Published:** 2020-12-07

**Authors:** Peter Tsvetkov, Nadav Myers, Julia Adler, Yosef Shaul

**Affiliations:** Department of Molecular Genetics, Weizmann Institute of Science, Rehovot 76100, Israel; nadav.myers@weizmann.ac.il (N.M.); julia.adler@weizmann.ac.il (J.A.)

**Keywords:** proteostasis, ubiquitin independent degradation, intrinsically disordered proteins, NADH-26S proteasome

## Abstract

The 26S proteasome is the endpoint of the ubiquitin- and ATP-dependent degradation pathway. Over the years, ATP was regarded as completely essential for 26S proteasome function due to its role in ubiquitin-signaling, substrate unfolding and ensuring its structural integrity. We have previously reported that physiological concentrations of NADH are efficient in replacing ATP to maintain the integrity of an enzymatically functional 26S PC. However, the substrate specificity of the NADH-stabilized 26S proteasome complex (26S PC) was never assessed. Here, we show that the binding of NADH to the 26S PC inhibits the ATP-dependent and ubiquitin-independent degradation of the structured ODC enzyme. Moreover, the NADH-stabilized 26S PC is efficient in degrading intrinsically disordered protein (IDP) substrates that might not require ATP-dependent unfolding, such as p27, Tau, c-Fos and more. In some cases, NADH-26S proteasomes were more efficient in processing IDPs than the ATP-26S PC. These results indicate that in vitro, physiological concentrations of NADH can alter the processivity of ATP-dependent 26S PC substrates such as ODC and, more importantly, the NADH-stabilized 26S PCs promote the efficient degradation of many IDPs. Thus, ATP-independent, NADH-dependent 26S proteasome activity exemplifies a new principle of how mitochondria might directly regulate 26S proteasome substrate specificity.

## 1. Introduction

The function of the 26S proteasome complex (26S PC) is considered to be completely dependent on ATP availability and hydrolysis [1,2,3]. This is largely due to the multiple roles of ATP in the process of ubiquitin-dependent degradation by the 26S PC. The 26S PC is composed of the 20S catalytic complex and the 19S regulatory particle that contains six ATPases, Psmc1-6, residing at the interface between the 19S and 20S PCs [4,5]. ATP binding and hydrolysis in the catalytic cycle of the 26S proteasome were shown to regulate the ubiquitin processing of the substrate, protein unfolding, and also to maintain the integrity of the 26S proteasomal complex [6,7]. In the absence of ATP, the 26S proteasome dissociates quite rapidly into the 20S and 19S particles [1,3,8]. However, 26S PC integrity is achieved also by CTP, UTP and ADP [1,9], by the unnatural nucleotides ATPγS and AMPPNP [10,11], and also by proteasome inhibitors [9]. In addition, proteins such as Ecm29 [12] act to stabilize 26S PC. As such, although the functions of the proteasome that require the hydrolysis of ATP cannot be replaced by other metabolites, the ATP function in stabilizing the 26S PC can be substituted by other natural and artificial metabolic molecules.

There is increasing evidence suggesting that altered cellular metabolism is associated with both proteasome function and integrity [13,14,15]. We have previously shown that NADH maintains 26S PC integrity in the absence of ATP. NADH is a key metabolic molecule that couples redox regulation to cellular metabolism, serving as both a shuttle of electrons from glycolysis and TCA cycle metabolism to the electron transfer chain, and also serving as a substrate for many antioxidant enzymes [16,17]. We have shown that NADH specifically interacts with a distinct NADH binding box in the N-terminus of Psmc1, a 19S AAA-ATPase subunit [18]. Furthermore, using differential sensitivity of NADH-26S PC to high levels of MgCl_2_, we showed that NADH-26S PC is detected in a number of mouse tissues. There are three distinct functions of ATP in 26S proteasome activity: maintaining stability, promoting ubiquitin processing and the unfolding of the substrates. This raises the possibility that proteins that are not ubiquitinated and do not need unfolding can be degraded by a stabilized and functional proteasome, even in the absence of ATP. Indeed, this was shown with ATPγS (an artificial non-hydrolysable form of ATP)-stabilized 26S PC, that could degrade disordered proteins such as casein, p21 and oxidized proteins [10,11,19].

About a third of eukaryotic proteins are intrinsically disordered proteins (IDPs) or consist of large disordered regions (IDRs) [20,21,22]. IDPs/IDRs, by large, are more labile proteins with short half-lives [23], and as many of them were shown to be degraded in an ATP-independent manner by the 20S proteasome [24,25,26,27], suggesting that at least a subset of IDPs do not require unfolding for proteasome degradation. In this work, we set out to determine how NADH affects 26S proteasome degradation and if NADH-stabilized 26S PCs can promote the ubiquitin-independent degradation of IDPs/IDRs.

## 2. Materials and Methods

### 2.1. Proteasomal Complex Stability Assay

We used the protocol previously reported by us [18], with minor modifications. In short, proteasomes from NIH3T3 cells or purified 26S proteasomes from rabbit muscles in Deg Buffer (50 mM Tris 7.5, 150 mM NaCl, 5 mM MgCl_2_) were supplemented with either ATP or NADH in the presence or absence of 8 mU/μL apyrase (Sigma, St. Louis, MO, USA). After incubation at 37 °C for the indicated time points, the reaction was loaded on nondenaturing 4% polyacrylamide gel for monitoring proteasomal complex stability.

### 2.2. Nondenaturing PAGE

Proteasomal samples were loaded on a nondenaturing 4% polyacrylamide gel as previously reported [18]. After blotting to nitrocellulose membranes, immunoblotting was conducted using the indicated antibodies.

### 2.3. Proteasomal Activity

To measure proteasomal activity, the hydrolysis of Suc-LLVY-AMC was quantified as described in the manufacturer’s protocol (Biomol, USA).

### 2.4. ^35^S In Vitro Translated Proteins and Purification

In vitro translation ^35^S Methionine labeled flag-Yap1, flag-Taz, flag-c-Fos and ODC were subjected to immunoprecipitation with flag-beads (Sigma) in Deg buffer, as previously reported [28]. After 1 h incubation at 4 °C, the flag beads were washed three times with Deg buffer. The proteins retained on the beads were eluted with the addition of Deg buffer containing 100 μg/mL of flag peptide (Sigma) and incubation at 37 °C for 20 min.

### 2.5. In Vitro Degradation Assay

In Vitro translated ^35^S Methionine-labeled either crude or purified proteins, and recombinant proteins were incubated in the presence or absence of 1 μg of purified 26S proteasomes, as previously described [24,28]. The reactions were conducted for the indicated times at 37 °C with the supplementation of either 2 mM ATP, ATPγS or NADH. Reactions were stopped with the addition of Laemmli sample buffer and heated at 95 °C for 5 min. The products were separated by polyacrylamide-SDS gel. ^35^S Methionine-labeled proteins were detected by autoradiography. The purified proteins were transferred to nitrocellulose membranes and detected with the indicated antibodies.

## 3. Results

### 3.1. NADH Inhibits ATP-Dependent, 26S Proteasome Degradation of ODC

**Figure 1:**
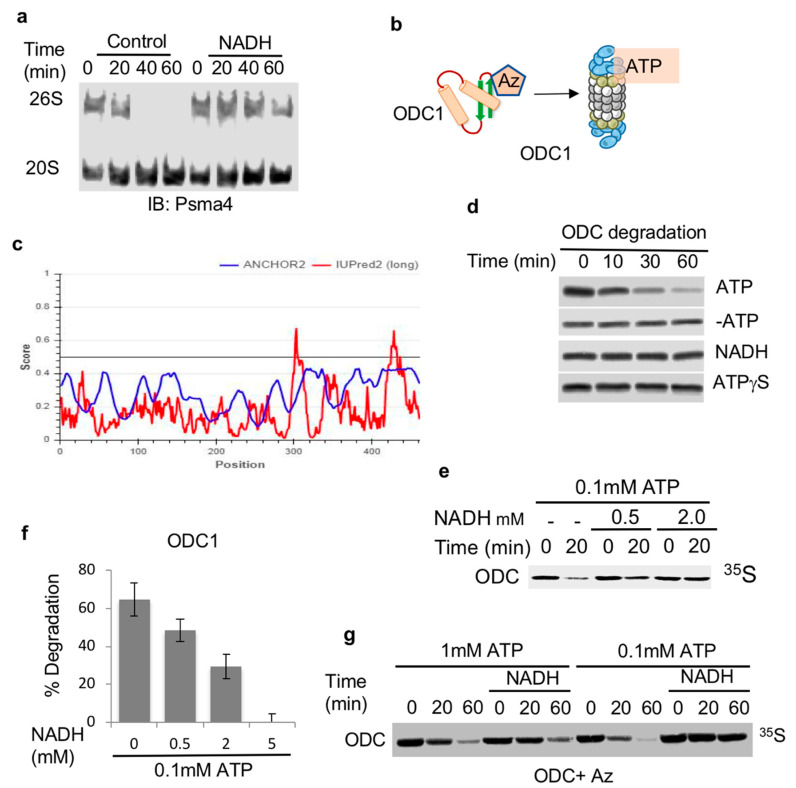
ODC is degraded by ATP-26S but not by NADH-26S proteasome. (**a**) NADH stabilizes the 26S PC. Proteasomes fractionated from NIH3T3 cells were incubated at 37 °C for indicated times in the presence or absence of 2 mM NADH. (**b**) Scheme/model (**c**) ODC (UniProt P11926) is, by large, an ordered protein based on the scores of prediction output using IUPred2A (the red curve) and ANCHOR2 (the blue curve) prediction programs. (**d**) NADH-26S proteasomes cannot induce ODC degradation by Az. ODC degradation in rabbit reticulocyte extract by Az was examined in the presence or absence of 2 mM ATP and an ATP-regenerating system (ATP). This reaction was also conducted under removal of the ATP by Apyrase (-ATP). Similar reactions were conducted in the presence of 2 mM NADH with Apyrase (NADH) and 2 mM ATPγS with Apyrase (ATPγS). (**e**) NADH represses ODC degradation. In vitro translated ^35^S Methionine-labeled ODC was subjected to degradation in reticulocyte lysate in the presence of Antizyme (Az). ODC degradation was examined in the presence of 0.1 mM ATP and increasing concentrations of NADH at 37 °C for 20 min. (**f**) The data from at least three independent experiments were averaged and shown with their standard deviation. (**g**) The NADH-mediated inhibition of ODC degradation is alleviated by higher ATP concentration. ODC degradation was examined in the presence of 1 mM or 0.1 mM ATP in the presence or absence of 5 mM NADH for the indicated time points at 37 °C. The level of ^35^S-labeled ODC degradation was visualized by autoradiography following SDS-PAGE and quantified.

Previously, we have reported that the 26S proteasome can be stabilized by NADH [18] and (Figure 1a). However, the functionality of such NADH-stabilized 26S proteasome was not determined. As such, we decided to systematically address the role of NADH in the regulation of 26S proteasome substrate degradation (Figure 1b). To uncouple the degradation process by the 26S PC from the ubiquitin regulation process, we utilized the antizyme (Az)-mediated ODC degradation system that has been extensively characterized in the context of ubiquitin-independent degradation by the 26S proteasome [29]. Az binds ODC monomerizes and targets ODC monomer to the 26S PC in the process of initiating ATP-dependent 26S proteasomal degradation (Figure 1c) [30,31]. ^35^S-Methionine labeled ODC was incubated with Az in rabbit reticulocyte extracts reported to support ODC degradation. As expected, ODC was completely degraded with the supplementation of ATP to the mix (Figure 1d). In the absence of ATP or in the presence of a non-hydrolysable form of ATP, ATPγS, no Az-mediated degradation of ODC was observed (Figure 1d). The supplementation of NADH in the absence of ATP was not sufficient to induce ODC degradation. However, when NADH was added in the presence of ATP, it resulted in the inhibition of Az-mediated degradation of ODC in a dose-dependent manner (Figure 1e,f). The inhibitory effect of NADH was alleviated by increasing amounts of ATP (Figure 1g). Thus, NADH binding to the 26S proteasome has an inhibitory effect on ubiquitin-independent degradation of ODC, possibly by generating NADH-26S PC.

### 3.2. ATPγS-26S PC Promotes the Degradation of IDPs

The unfolding of globular proteins for their degradation is mediated by hydrolysis of ATP by the 26S PC ATPase subunits [7]. ATPγS is a form of ATP that is almost non-hydrolysable, and as such inhibits the proteasome functions that rely on the ATP hydrolysis of ATP such as protein unfolding. Some unfolded and denatured proteins can be degraded by the ATPγS-stabilized 26S PC (in the absence of ATP) [11]. Here, we experimentally addressed the question whether IDPs, which inherently lack a defined structure and thus are independent of the unfolding step during degradation, can be degraded by the ATPγS 26S PC.

**Figure 2:**
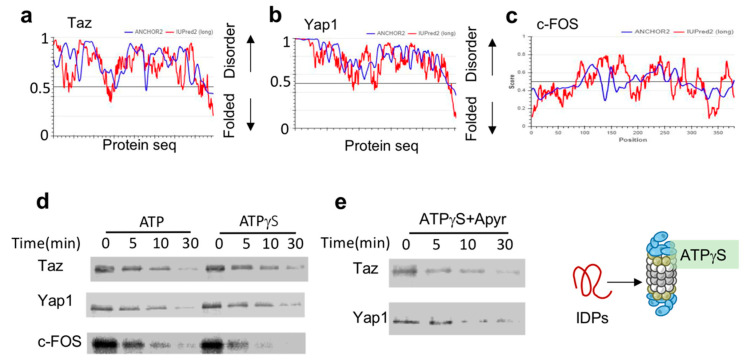
IDPs are degraded by ATPγS 26S PC (a to c). (**a**) Taz (UniProt Q4V7E6). (**b**) Yap (UniProt P146937) and (**c**) c-Fos (UniProt P01100), are intrinsically disordered based on the scores of prediction output using IUPred2A (the red curve) and ANCHOR2 (the blue curve) prediction programs. (**d**) Taz, Yap1 and c-Fos are degraded by ATPγS 26S PC. In vitro translated ^35^S Methionine labeled and purified Taz, Yap1 and c-Fos were subjected to degradation by the 26S proteasome in the presence or absence of either 2 mM ATP or ATPγS. (**e**) In the presence of ATPγS the elimination of residual ATP by preincubation with 5 mu/μL apyrase did not inhibit the 26S ability to degrade Taz and Yap1.

The key transcription regulators Taz, Yap1 and c-Fos are all predicted to be highly disordered (Figure 2a–c). To test their susceptibility to ATPγS-26S PC, we in-vitro translated and ^35^S-Metionine-labeled flag-tagged versions of these IDPs. To prevent any undesired effects that can arise from the reticulocyte mix, we further immune-purified these proteins by flag affinity purification followed by flag peptide elution, resulting in purified, ^35^S-Methionine-labeled IDPs. Each of these IDPs was separately incubated with either ATP-26S or ATPγS-26S PCs. ATPγS-stabilized 26S PC was efficient in degrading all three tested IDPs (Taz, Yap, c-Fos) (Figure 2d). This was not due to residual ATP in the ATPγS 26S PC, as degradation of Yap1 and Taz (Figure 2e) remained efficient even in the presence of apyrase, an enzyme that converts ATP and ADP to AMP [9,10,18]. The ability of the ATPγS-26S PC to mediate IDP degradation is consistent with the reports that ATPγS mediates degradation of proteins lacking a defined structure [10,11,32], and further suggest that hydrolysable ATP is not required for IDP degradation by the 26S PC.

### 3.3. NADH-26S Proteasomes Can Degrade IDPs

**Figure 3:**
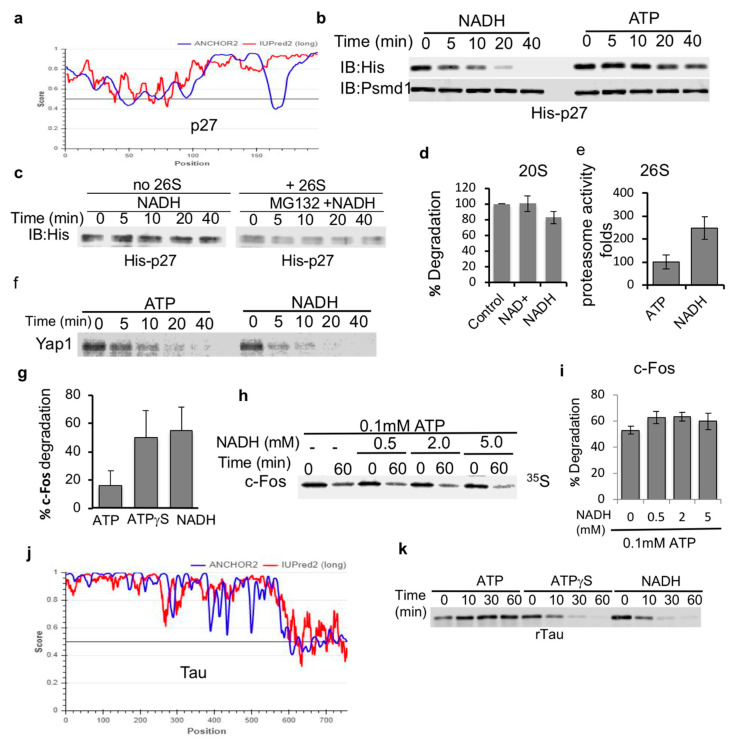
IDPs are susceptible to NADH-26S PC degradation. (**a**) p27 (UniProt P46527) is a disordered protein based on the prediction output using IUPred2A (red curve) and ANCHOR2 (blue curve) prediction programs. (**b**) Recombinant p27 is susceptible to degradation by the NADH-26S proteasome. Purified recombinant p27 protein was subjected to 26S proteasomal degradation. Time kinetics were analyzed for p27 degradation in the presence of either 2 mM ATP or NADH. His-p27 was detected by immunoblotting with anti-His antibody. (**c**) p27 is stable in the presence of NADH alone or in the presence of 26S proteasomes, 2 mM NADH and 25 μM of the proteasomal inhibitor MG132. (**d**) NADH has minor inhibitory effect on the purified 20S proteasome mediated degradation of synthetic substrate of the Chymotrypsin-like activity as measured based on the hydrolysis of Suc-LLVY-AMC substrate. (**e**) NADH induces the 26S proteasomal activity as measured by the cleavage of the Suc-LLVY-AMC peptide by purified 26S proteasomes in the presence of either 1 mM ATP or NADH. (**f**) In vitro translated purified proteins are degraded by the NADH-26S proteasome. In vitro translated ^35^S Methionine labeled flag-Yap1 was purified by flag IP followed by flag elution in the degradation buffer (see Section 2). Yap1 degradation kinetics by the 26S proteasome were analyzed in the presence of either 2 mM NADH or ATP. (**g**) Degradation of purified c-Fos was analyzed following 30 min incubation with 26S proteasomes in the presence of 2 mM ATP, ATPγS or NADH. (**h**) In vitro translated ^35^S Methionine-Fos degradation was examined in the presence of 0.1 mM ATP and increasing concentrations of NADH at 37 °C for 60 min. (**i**) The data from at least three independent experiments, as shown in (**h**) were averaged and shown with their standard deviation. (**j**) Tau (UniProt P10636) is a highly disordered protein as demonstrated by the scores of prediction output using IUPred2A (red curve) and ANCHOR2 (blue curve) prediction programs. (**k**) NADH and ATPγS induce 26S proteasomal degradation of recombinant tau protein (rTau) degradation by the 26S proteasome. Degradation was examined in the presence of either 2 mM ATP, ATPγS or NADH.

Next, we set out to determine if NADH-stabilized 26S PC are capable of facilitating the degradation of IDPs. Initially, we used bacterially expressed and purified p27, a highly disordered protein [33,34] (Figure 3a). NADH 26S-PC were very efficient at promoting the degradation of p27, kinetically even faster than ATP-26S PC (Figure 3b). To validate that the destabilization of p27 is due to proteasome-mediated degradation, we incubated p27 either alone or in the presence of the proteasome and a proteasome inhibitor MG132. As expected, MG132 completely blocked the degradation of p27 by the NADH-26S proteasome (Figure 3c). In our degradation reactions, NADH was not oxidized (data not shown) validating that NADH is a 26S PC-stabilizing cofactor and not the substrate of an unknown enzyme that facilitates proteasome activity. NADH (and NAD^+^) also did not have an effect on the catalytic activity of purified 20S proteasomes (Figure 3d). However, NADH induced an increase in the catalytic activity of the 26S proteasome (Figure 3e). These results suggest that NADH does not directly affect the catalytic activity of the proteasome but possibly affects the gating of the 26S proteasome complex. Together, these data suggest that NADH-26S PC is active in degradation of p27 IDP.

To generalize the proposed model of IDP being highly efficiently degraded by the NADH-26S PC, we examined the in vitro translated and purified ^35^S-Methionine-labeled Yap and c-Fos proteins. Yap protein was efficiently degraded by the NADH-26S PC as compared to ATP-26S PC (Figure 3f). Purified in vitro translated ^35^S-Methionine c-Fos was also efficiently degraded by NADH-26S PC with similar time kinetics of ATPγS-26S, but more efficiently than ATP-26S activity (Figure 3g). The observation that ATPγS is more active than ATP in inducing degradation of IDPs by the 26S PC is consistent with the published reports [11]. Unlike ODC, c-Fos degradation was not inhibited by increasing concentration of NADH (Figure 3h,i). We further analyzed the bacterially expressed and purified tau protein as another highly disordered protein substrate (Figure 3j). Tau protein was efficiently degraded by both NADH- and ATPγS- 26S PCs but was not efficiently processed by ATP-26S PC (Figure 3k). Taken together, our data indicate that IDPs are highly susceptible to degradation by the NADH-26S PC in a similar fashion as observed for ATPγS-26S PC and, in some cases, the degradation of IDPs by the NADH-PC is more efficient than that observed for ATP-26S PC.

## 4. Discussion

We show here that NADH can directly regulate substrate specificity of the 26S proteasome in vitro. Our findings here elaborate on our previous observation, showing that functional 26S proteasome can be stabilized by NADH [18]. As there is no straightforward way to distinguish the functionality of NADH-26S from the ATP-26S PC in the context of the cell, we chose a reductive in vitro approach to test our hypothesis that NADH-26S PC is capable of facilitating the degradation of proteins that do not require the ATP-dependent functions of the proteasome such as ubiquitin processing and unfolding. As such, intrinsically disordered proteins (IDPs) are the perfect candidates, as many of them are readily degraded by the 20S CP in vitro [27,28]. We show here that IDPs are also substrates of this new form of 26S PC, namely the NADH-26S PC.

Our findings illuminate that NADH is a regulator of proteasome function, as it can promote the degradation of IDPs but also inhibit the natural function of ATP-dependent degradation of the 26S proteasome, a function that can affect substrate specificity in the context of the cell. The possibility that NADH competes for ATP-binding sites, resulting in competitive inhibition of the ATP-dependent functions of the 26S PC, was ruled out [18]. The other possibility is that NADH binds the N-terminus of PSMC1 (as previously shown) and possibly other 19S subunits, inducing an allosteric effect that results in inhibition of the directional cycling of the ATP in the hexametric ATPase ring [18]. Recent structural analysis of the ATPγS-26S reveals a strong structural rearrangement of many of the 19S subunits in the ATPγS-bound state, resulting in higher alignment of the ATPase ring with the gate of the 20S core particle, suggesting a state with facilitated translocation of the substrate [35]. This is in agreement with what we observed here with ATPγS-26S degradation of IDPS. The possibility that NADH exerts a similar allosteric structural shift can be addressed with Cryo-EM analysis in the future. However, many of the predicted NADH binding motifs in the 19S subunits are conserved in vertebrates, but not in yeast [18].

The emerging picture is that a non-hydrolysable ATP is sufficient for 26S complex formation/stabilization, whereas ATP hydrolysis is essential for the process of unfolding of the structured substrates [7,10,11,32,36,37]. The finding that IDPs are degraded by the 26S PC in the absence of ATP hydrolysis lends further support to this model. Interestingly however, we also observed that ATP-26S PC was actually inefficient in degrading certain IDPs. This might mean that ATP has a role in allosterically gating the 20S catalytic complex or that the binding of ATP masks certain IDP to target the 26S proteasome.

**Figure 4:**
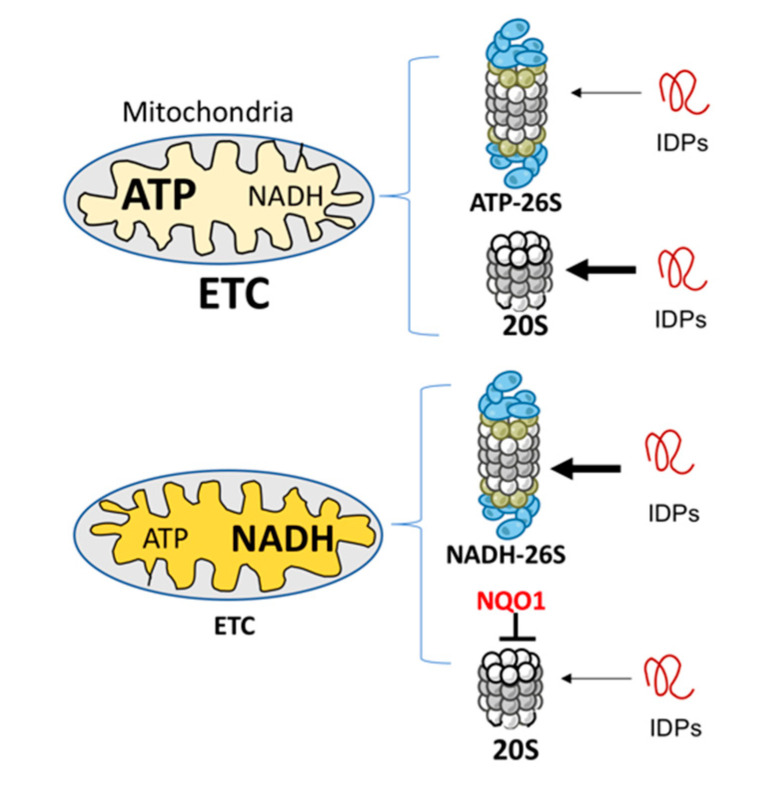
Mitochondria physiology and IDP degradation. Mitochondria with efficient electron transfer chain (ETC) activity (**upper**) generate a high level of ATP and low level of NADH. An opposite picture is obtained with mitochondria with low, or malfunctioning ETC (**lower**). The ATP and NADH levels regulate the formation of different types of 26S PC. Under high levels of NADH, NADH-26S PC is formed that is active in IDP degradation (the thick arrow). However, NADH through NQO1 has a repressive function in inhibiting IDP degradation by the 20S proteasome [42].

Whether NADH-26S PC has a physiological role is an important question. Analyzing the effect of various concentrations of ATP and NADH on IDP and ODC degradation led us to conclude that the formation of NADH-26S PC is reversible and depends on the NADH/ATP ratio. Under normal ATP concentrations at 1–10 mM [38], the NADH concentration of 10–100 μM is required to efficiently form the NADH-26S PC. These physiological concentrations suggest that, in the cells, a certain fraction of the 26S PC is of the NADH type. Cellular NADH level is determined by the electron transfer chain (ETC) functionality [14,39]. Inefficient ETC activity results in higher NADH levels and lower levels of ATP-26S PC [14], and under this condition, the NADH-26S PC level is expected to increase. Under this condition, mitochondrial biogenesis is compromised by NADH-dependent degradation of PGC-1α, a transcription co-activator regulating mitochondrial biogenesis [40]. NADH also has an indirect role in inhibiting 20S PC-mediated IDP degradation via NQO1 [41]. Overall, the emerging picture is that defective ETC remodels the IDP degradation process to be more prone to NADH 26S PC degradation (Figure 4).

## 5. Conclusions

Proteasomal degradation of intrinsically disordered proteins (IDPs) or proteins consist of large disordered regions (IDRs) is not exclusively mediated via the classical ubiquitin-26S proteasome pathway but also subjected to ubiquitin-independent degradation. Here we investigated the process of IDP/IDR degradation via unique class of 26S proteasome that is free of ATP. Two different such 26S proteasomes were investigated; the non-hydrolysable ATPγS-26S and the recently reported NADH-26S proteasomes. We show here that both are in vitro active in degradation of IDP/IDR but not of ODC structured protein. The finding that physiological metabolite like NADH uniquely regulates IDP/IDR degradation exemplifies a new principle of how mitochondria, the key organelle in NADH production, regulate IDP/IDR homeostasis.
